# Underrecognized Tick-Borne Encephalitis in Serbia: Evidence from Patients with Suspected West Nile Virus Neuroinvasive Disease

**DOI:** 10.3390/pathogens15060587

**Published:** 2026-05-29

**Authors:** Dragana Mijatović, Ana Marija Radevska, Dejan Jakimovski, Lidija Popović-Dragonjić, Biljana Popovska Jovičić, Jagoda Gavrilović, Siniša Sević, Dajana Lendak, Irina Stojanac, Alejandro Cabezas-Cruz, Andreas Pilz, Tomás Cervantes Rincón, Jasmine Oberti-Cantergiani, Davide F. Robbiani, Pavle Banović

**Affiliations:** 1 Diagnostics and Laboratory Research Task Force, Balkan Association for Vector-Borne Diseases, 21000 Novi Sad, Serbia; draganav77@gmail.com; 2 Department of Microbiology, Pasteur Institute Novi Sad, 21000 Novi Sad, Serbia; 3 Epidemiology and Biostatistics Task Force, Balkan Association for Vector-Borne Diseases, 21000 Novi Sad, Serbia; anamarija_radevska@yahoo.com; 4 Clinical Medicine Task Force, Balkan Association for Vector-Borne Diseases, 21000 Novi Sad, Serbia; dejan.jakimovski@medf.ukim.edu.mk (D.J.); lidija_popovic2003@yahoo.com (L.P.-D.); biljanapop@yahoo.com (B.P.J.); jgavrilovic@outlook.com (J.G.); 5 University Clinic for Infectious Diseases and Febrile Conditions, 1000 Skopje, North Macedonia; 6 Clinic for Infectology, University Clinical Center, 18000 Niš, Serbia; 7 Department of Infectious Diseases and Epidemiology, Faculty of Medicine, University of Niš, 18000 Niš, Serbia; 8 Clinic for Infectology, University Clinical Centre Kragujevac, 34000 Kragujevac, Serbia; 9 Department of Infectious Diseases, Faculty of Medical Sciences, University of Kragujevac, 34000 Kragujevac, Serbia; 10 Clinic for Infectious Diseases, University Clinical Center of Vojvodina, 21000 Novi Sad, Serbia; sinisa.sevic@mf.uns.ac.rs (S.S.); dajana.lendak@mf.uns.ac.rs (D.L.); 11 Department of Infectious Diseases, Faculty of Medicine in Novi Sad, University of Novi Sad, 21000 Novi Sad, Serbia; 12 Clinic for Neurology, University Clinical Center of Vojvodina, 21000 Novi Sad, Serbia; stojanacirina96@gmail.com; 13 Anses, INRAE, Ecole Nationale Vétérinaire d’Alfort, UMR BIPAR, Laboratoire de Santé Animale, F-94700 Maisons-Alfort, France; alejandro.cabezas@vet-alfort.fr; 14 Vaccines, Antivirals, and Evidence Generation, Pfizer Biopharma, 2304 Vienna, Austria; andreas.pilz@pfizer.com; 15 Institute for Research in Biomedicine, Università della Svizzera Italiana, 6500 Bellinzona, Switzerland; tomas.cervantes@irb.usi.ch (T.C.R.); jasmine.cantergiani@irb.usi.ch (J.O.-C.);; 16 Clinic for Lyme Borreliosis and Other Tick-Borne Diseases, Department of Prevention of Rabies and Other Infectious Diseases, Pasteur Institute Novi Sad, 21000 Novi Sad, Serbia; 17 Department of Microbiology with Parasitology and Immunology, Faculty of Medicine in Novi Sad, University of Novi Sad, 21000 Novi Sad, Serbia

**Keywords:** tick-borne encephalitis virus, West Nile virus, neuroinvasive disease, orthoflavivirus, Serbia, neutralization assay, serology, underdiagnosis

## Abstract

Tick-borne encephalitis (TBE) is an emerging vector-borne disease in Europe, but its epidemiology remains poorly defined in Serbia. In orthoflavivirus-endemic settings, diagnostic challenges may contribute to underrecognition of TBE, particularly among patients with suspected West Nile virus (WNV) infection. We conducted a multicenter retrospective study including patients hospitalized between 2018 and 2023 with suspected WNV neuroinvasive disease or viral encephalitis of unknown etiology. Serum samples were tested for TBEV-neutralizing antibodies using a microneutralization assay. Among 79 patients, TBEV-neutralizing antibodies were detected in four (5.1%). Most reactive cases occurred in patients initially classified as having suspected WNV-associated meningoencephalitis, while TBE had not been considered in the differential diagnosis at admission. These findings suggest that TBE may be underrecognized in Serbia and highlight the importance of confirmatory testing in orthoflavivirus-endemic settings. Strengthening clinical awareness and surveillance will be essential to better define the burden of TBE and inform prevention strategies.

## 1. Introduction

Tick-borne encephalitis (TBE) is an emerging, vaccine-preventable vector-borne disease caused by tick-borne encephalitis virus (TBEV; *Orthoflavivirus encephalitidis*). Despite the availability of effective vaccines, TBE remains a major public health concern across Eurasia [1]. The virus is primarily transmitted by hard ticks, with *Ixodes ricinus* serving as the main vector in Europe [2]. In contrast to mosquito-borne orthoflaviviruses such as West Nile virus (WNV; *Orthoflavivirus nilense*), which are maintained in bird–mosquito transmission cycles [3], TBEV is maintained in tick–vertebrate host systems, reflecting fundamentally different ecological and transmission dynamics. Humans are incidental, dead-end hosts who become infected after exposure within natural TBEV foci, most often via the bite of an infected *I. ricinus* tick and occasionally through ingestion of contaminated, unpasteurized milk or dairy products [4,5,6].

In humans, TBEV infection is predominantly asymptomatic (i.e., subclinical). Although the frequency of asymptomatic infection is difficult to quantify precisely, it is estimated that 70–98% of exposed individuals do not develop clinically apparent disease [7,8]. In symptomatic cases, infection usually begins with a nonspecific febrile illness accompanied by influenza-like symptoms. In most patients, viral clearance occurs within approximately 7 days, and no further disease progression is observed. However, in approximately 30% of symptomatic individuals, the initial phase is followed by a symptom-free interval of 4–10 days, after which a second phase develops with signs of central nervous system (CNS) involvement, corresponding to the neurological phase of TBE [9,10]. Long-term neurological sequelae are frequently reported even in patients who recover from the acute illness [1,11,12].

Surveillance of TBE is not uniform across Europe [1,13,14]. In 2022, 20 countries of the European Union reported 3650 TBE cases, of which 3516 (96.3%) fulfilled the confirmed case definition [15]. This represented a 14% increase in the reporting rate compared with 2021 and may reflect improved surveillance, increased awareness, enhanced diagnostic capacity, greater exposure of the European population, or a combination of these factors. Clinical awareness of TBE, particularly among healthcare professionals, is therefore a critical component of effective surveillance and accurate case detection. Serbia provides a relevant example: despite TBE being a notifiable communicable disease, awareness among medical professionals has been reported to be insufficient [16]. This may contribute to underrecognition and underreporting, as only 39 TBE cases were officially reported by the Public Health Institute (PHI) of Serbia between 2004 and 2023 [17,18], despite recent seroprevalence findings showing TBEV-neutralizing antibodies in 3/450 (0.66%) tick-infested individuals [19] and TBEV-reactive antibodies in 3/15 (20%) patients discharged with a diagnosis of viral meningitis and/or viral encephalitis of unknown etiology [20]. In 2024, PHI of Serbia launched a 4-month pilot TBE surveillance program, but unfortunately these results have not yet been published. Few field studies aimed to detect TBEV foci in Serbia, but with limited success, as no foci were identified [21,22]. Only recently, human infections could be extrapolated from seroprevalence studies, which found TBEV-neutralizing antibodies in individuals who were infested by ticks in rural areas of Fruška Gora, Divčibare, and Bukulja mountains [19].

Because no specific antiviral therapy for TBE is available, vaccination is of paramount importance [23]. However, Serbia remains one of the few European countries where TBE vaccines are not available [24], despite official recommendations for their use in persons at risk of TBEV exposure [25]. Beyond insufficient awareness among healthcare professionals, this may also reflect diagnostic challenges, as TBE diagnosis in Serbia relies predominantly on ELISA-based testing [26], whereas confirmatory neutralization assays have only been available since 2022. These limitations are particularly important in a country endemic for another orthoflavivirus causing neurotropic disease (WNV), which is recognized as a major public health threat [27]. ELISA-based assays may have reduced specificity because of serologic cross-reactivity, increasing the risk that TBE cases are misclassified as WNV infections [28,29]. This is especially likely during the summer season, when WNV activity is heightened and clinicians may be less inclined to consider TBEV infection in the differential diagnosis.

Although no official data on the incidence of TBE in Serbia are currently available, previous studies suggest that TBEV infections occur in the Serbian population [19]. In this context, the aim of the present study was to retrospectively assess serological evidence of TBEV exposure in patients hospitalized between 2018 and 2023 at three University Clinical Centers in Serbia, who were clinically suspected of having WNV infection with CNS involvement or viral encephalitis of unknown etiology.

## 2. Materials and Methods

### 2.1. Study Design and Patient Selection

Eligible patients were those who presented at the Clinics for Infectious Diseases of Niš, Novi Sad, or Kragujevac between 2018 and 2023 (*n* = 82; [30,31] and unpublished) with either suspected WNV infection involving CNS or viral encephalitis of unknown etiology, based on signs, symptoms, and available laboratory findings up to at least 7 days after hospitalization. Patients with confirmed bacterial CNS infection or confirmed viral CNS infection of non-orthoflavivirus etiology were excluded from the study. Suspected WNV infection was clinically defined by the presence of encephalitis and/or meningitis and/or fever ≥38 °C in combination with detection of WNV-reactive IgG antibodies in serum by ELISA in laboratories affiliated with the respective clinical centers. Detection of anti-WNV IgG allows identification of prior exposure but does not distinguish between past and acute infection. Therefore, WNV IgG positivity alone was not considered definitive evidence of acute WNV infection, particularly in an orthoflavivirus-endemic setting where serologic cross-reactivity may occur. None of the enrolled individuals had been previously immunized against TBE or yellow fever based on own reporting. Travel history was not systematically recorded, limiting the assessment of potential exposure to other orthoflaviviruses. The informed consent was waived, 3 mL of blood was collected in BD Vacutainer^®^ SST™ Tubes (BD, Franklin Lakes, NJ, USA). Blood samples were allowed to clot at room temperature and centrifuged at 2000× *g* for 10 min. Serum was separated, aliquoted, and stored at −80 °C until analysis. Prior to testing, samples were heat-inactivated (56 °C for 1 h).

### 2.2. Serological Analysis

#### 2.2.1. ELISA Against the TBEV and WNV EDIII Proteins

Serum IgG binding to the envelope domain III (EDIII) recombinant protein of TBEV (TEBV_EDIII_) and WNV (WNV_EDIII_) was measured by enzyme-linked immunosorbent assay (ELISA) as previously described [30,31]. Briefly, clear flat-bottom 384-well plates (Thermo Fisher Scientific™, Waltham, MA, USA, Cat. No 464718) were coated overnight at room temperature with 50 ng/well with the EDIII protein in phosphate-buffered saline (PBS). After washing with PBS-T (PBS 0.05%—Tween-20), plates were blocked for 2 h at room temperature with PBS, 1 mM EDTA, 0.05%—Tween-20, and 1% bovine serum albumin (BSA). Following an additional wash with PBS-T, prediluted serum samples were added and incubated for 1 h at room temperature. Plates were then washed and incubated for 1 h at room temperature with horseradish peroxidase (HRP)-conjugated sheep anti-human IgG secondary antibody (VWR, NA933) diluted 1:5000 in PBS-T. After a final wash step, TMB (3,3′,5,5′-tetramethylbenzidine) substrate (Thermo Fisher Scientific™, Cat. No 34021) was added for signal development, and the reaction was stopped with 1 M sulfuric acid. Absorbance was measured at 450 nm using a BioTek Epoch2 microplate reader (BioTek, Winooski, VT, USA). Serum samples were serially diluted 5-fold two times starting at 1:50, and the area under the curve was calculated with GraphPad Prism 10 software.

#### 2.2.2. Production and Neutralization of WNV Reporter Viral Particles (RVPs)

WNV_RVPs_ were produced as previously described [30,31]. Briefly, 7.5 × 10^5^ HEK293T cells per well were seeded in 6-well plates in DMEM high glucose GlutaMAX (Gibco, Thermo Scientific™, Waltham, MA, USA, Cat. No 35050061) supplemented with 10% FBS, 100 U/mL penicillin, and 100 µg/mL streptomycin. After 24 h, cells were co-transfected with pWNV-NanoLuc and the WNV CprME expression plasmids at a 1:3 ratio with Lipofectamine 2000 (Invitrogen, Thermo Fisher Scientific™, Waltham, MA, USA, Cat. No 11668019). After 5 h the medium was replaced with DMEM high glucose GlutaMAX supplemented with 3% FBS, 100 U/mL penicillin, and 100 µg/mL streptomycin. Supernatants containing the RVPs were collected after 48 h, filtered through 0.22 µm membranes, aliquoted, and stored at −80 °C. Infectivity was determined by titration. For neutralization assays, 96-well plates were seeded with 1.5 × 10^4^ Huh-7.5 cells per well in 100 µL of DMEM high glucose GlutaMAX supplemented with 10% FBS, 1% sodium pyruvate, 1% non-essential amino acids, 100 U/mL penicillin, and 100 µg/mL streptomycin. After 24 h, serum samples were diluted in Ba-1 medium consisting of Medium 199 (Gibco, Thermo Scientific™, Waltham, MA, USA), 1% BSA, 100 U/mL penicillin, and 100 µg/mL streptomycin. Diluted serum (200 µL) was mixed with an equal volume of diluted RVPs and incubated for 1 h at 37 °C. An amount of 100 µL of the mixture was then added to the cells in triplicate and incubated overnight at 37 °C. After 24 h, supernatants were removed, cells were washed with PBS, and lysed in 50 µL lysis buffer (Promega, Madison, WI, USA, Cat. No E1531). NanoLuc activity was measured using the Nano-Glo Luciferase Assay System (Promega, Madison, WI, USA, Cat. No N1120) on a GloMax Discover System reader (Promega, Madison, WI, USA). Serum samples were tested at a 1:100 dilution.

#### 2.2.3. TBEV Microneutralization Assay

The TBEV strain Neudörfl (National Collection of Pathogenic Viruses, Wiltshire, UK; Cat. No 0201139v) was cultured in a biosafety level 2+ Laboratory for Vector Borne Pathogens at Pasteur Institute Novi Sad using a monolayer of BHK-21/C13 cells (BS CL 8, Istituto Zooprofilattico Sperimentale, Brescia, Italy). Virus stocks were prepared at a concentration of 100 Tissue Culture Infectious Dose (TCID)/100 μL and stored at −80 °C. The microneutralization test (micro-NT) was conducted using a 96-well cell culture plate (Thermo Scientific™, Waltham, MA, USA, Cat. No 130338). Detailed TBEV-microneutralization procedure was provided previously [19]. A summary of key assay parameters, including controls and interpretation criteria, is provided here to facilitate reproducibility. Briefly, samples were serially diluted in two replicates, from 1:5 to 1:40 and mixed with 100 TCID50 units of TBEV. If the sample was neutralizing the virus at 1:40 dilution, a second assay was performed with two-fold serial dilutions from 1:40 to 1:1280 (i.e., wide titration). Back titration of the TBEV was performed in every assay. A sample was considered positive if the TBEV titer was ≥1:10.

### 2.3. Statistical Analysis

Statistical analysis was performed using GraphPad Prism version 10 (GraphPad Software, San Diego, CA, USA). Categorical variables were summarized as counts and percentages, while continuous variables were expressed as mean and range. Patients were categorized into four age groups: ≤30 years (young individuals), 31–50 years (middle-aged adults), 51–64 years (older adults), and ≥65 years (elderly). This stratification was adapted to facilitate epidemiologically meaningful subgroup comparisons while preserving adequate sample sizes within each category and accounting for potential age-related differences in tick exposure patterns. Associations between categorical variables were evaluated using the χ^2^ test or Fisher’s exact test, as appropriate. Due to small sample sizes in certain subgroups, Fisher’s exact test was used for 2 × 2 comparisons. A *p*-value of <0.05 was considered statistically significant. No correction for multiple comparisons was applied, given the limited number of analyses. Given the limited number of TBEV-reactive cases, statistical analyses were considered exploratory and interpreted primarily in a descriptive context.

## 3. Results

### 3.1. Study Population Characteristics

During the study period from 2018 to 2023, a total of 82 patients were hospitalized at University Clinical Centers of Niš, Kragujevac and Novi Sad. Three serum samples were unsuitable for testing due to toxicity for cell lines and were excluded from the analysis, resulting in a final cohort of 79 patients. The study population included 52/79 males (65.8%) and 27/79 females (34.2%). The mean age of the patients was 58.7 years (range: 16–88 years). Most patients were classified as elderly (35/79; 44.3%), followed by older adults (23/79; 29.1%), middle-aged adults (11/79; 13.9%), and young individuals (10/79; 12.7%).

### 3.2. Clinical Presentation and Initial Diagnoses

**Figure 1:**
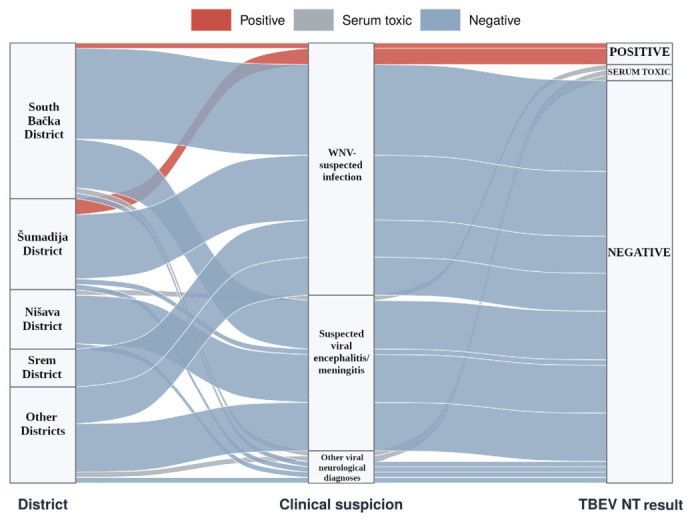
Sankey diagram connecting municipality of residence to reported diagnosis and TBEV neutralization results for the 82 patients of this study. Flow width is proportional to patient number. Diagnoses were harmonized into broader categories for clarity.

**Figure 2:**
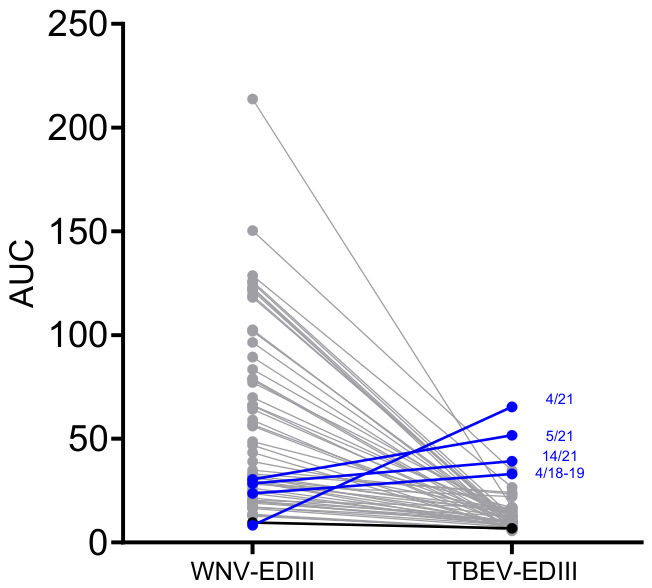
IgG binding to the EDIII of WNV and TBEV was measured by ELISA. Binding is shown as the area under the curve (AUC) calculated from serial serum dilution–response curves. Higher AUC values indicate stronger cumulative IgG binding across the tested dilutions. For each serum, WNVEDIII and TBEVEDIII reactivity were compared to identify the dominant binding pattern. WNVEDIII reactivity is shown in grey, TBEVEDIII reactivity is shown for comparison, and the four samples with higher TBEVEDIII than WNVEDIII reactivity are highlighted in blue. The negative serum control is shown in black. AUC-based binding results indicate relative antigen reactivity and require confirmatory testing for etiologic interpretation.

Patients were primarily treated under the following diagnoses: suspected viral encephalitis (25/79; 31.6%), viral meningoencephalitis (WNV) (25/79; 31.6%), and viral encephalitis (WNV) (20/79; 25.3%), followed by suspected viral meningoencephalitis (3/79; 3.8%) (Figure 1). Other diagnoses, such as encephalopathy, West Nile fever, polyradiculoneuritis, suspected viral encephalomyelitis, and WNV meningitis occurred only once (1/79; 1.26%) (Figure 2). Serum samples were obtained at a mean of 12.9 days after symptom onset (median 11.5 days; IQR: 8–17 days).

### 3.3. Serum IgG Binding to WNV and TBEV EDIII in ELISA

Patient sera (*n* = 79) were evaluated for the presence of antibodies binding to the virus EDIII in ELISA (Figure 2). In this visualization, each point represents the cumulative ELISA binding signal across the tested serum dilutions; higher AUC values indicate stronger IgG binding to the corresponding EDIII antigen, but AUC values alone should not be interpreted as proof of acute infection. EDIII-based ELISAs were previously shown to help differentiate antibody responses to different orthoflaviviruses [30,31], and binding was represented as the area under the curve (AUC) calculated from the serum dilution–response curve, showing IgG reactivity across the tested dilutions. Most samples were positive for WNV_EDIII_ IgG, displaying higher reactivity than to the TBEV_EDIII_ antigen, which was used alongside for comparison (grey). Unexpectedly, four samples showed the opposite pattern, with higher IgG reactivity for TBEV_EDIII_ (blue).

### 3.4. Serum Neutralization of WNV Reporter Virus Particles

**Figure 3:**
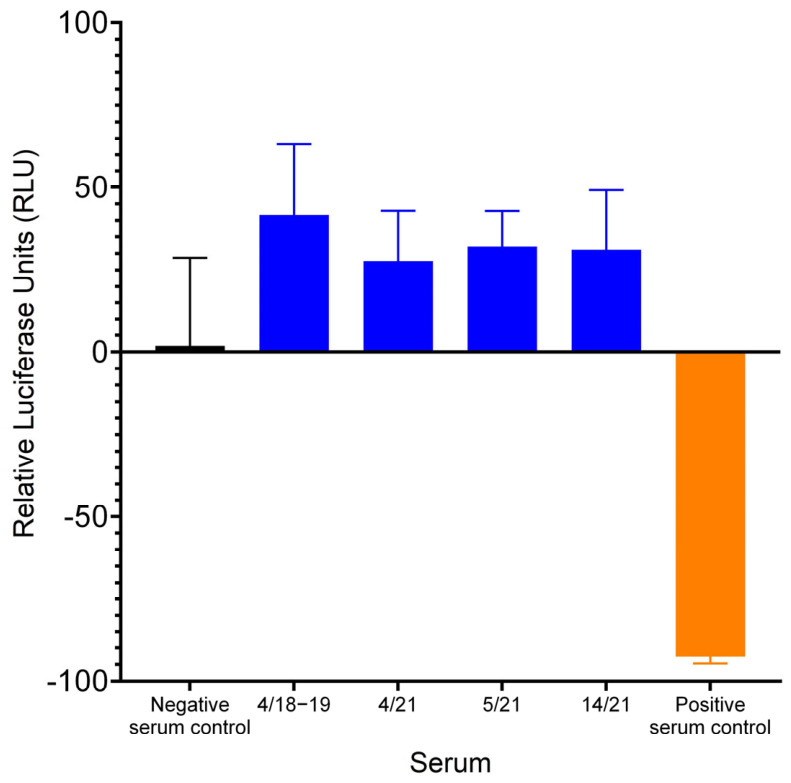
Neutralization of WNV reporter virus particles (WNVRVPs). Sera were tested at a 1:100 dilution for their ability to neutralize WNVRVPs encoding luciferase. Relative luciferase units (RLU) reflect WNVRVP infection of target cells; lower RLU values indicate stronger neutralization. The four sera with preferential TBEVEDIII IgG binding in Figure 2 are shown in blue also in this figure. Negative serum is shown in black, and serum from a WNV-infected individual is shown in orange as a positive control.

To confirm the lack of robust reactivity to WNV_EDIII_, the four sera positive for TBEV_EDIII_ IgG were next tested for neutralization of WNV_RVPs_ encoding for luciferase (Figure 3). In this assay, neutralization results in a decrease of luciferase signal. In agreement with the ELISA results, all four sera failed to neutralize WNV_RVPs_. Preferential TBEV EDIII binding and absence of detectable WNV neutralization support non-WNV orthoflavivirus antibody reactivity, most compatible with prior TBEV or related tick-borne orthoflavivirus exposure; however, these findings do not establish TBEV as the cause of the acute neurological syndrome.

### 3.5. Serum Neutralization of Authentic TBEV

TBEV neutralization was performed next on all 79 samples using authentic virus by micro-NT. Only the four samples (4/79; 5.1% of total) that were reactive to TBEV_EDIII_ in ELISA displayed TBEV neutralizing activity. In patients where TBEV-neutralizing antibodies were found, TBEV titers were 1:10 (2/4; 50%), 1:20 (1/4; 25%), and 1:640 (1/4; 25%) (Table 1).

### 3.6. Demographic Distribution of TBEV-Reactive Cases

The distribution of TBEV-neutralizing antibodies was equal between males and females (2/4; 50% each). The mean age of patients with TBEV-neutralizing antibodies was 72.5 years (range: 55–81 years), with most cases occurring among elderly individuals (3/4; 75%), while no reactive cases were identified among young or middle-aged patients. However, no statistically significant association was observed between age group and TBEV reactivity (χ^2^ = 2.041, df = 3, *p* = 0.564); thus, given the sample size, these analyses are best interpreted as descriptive. Notably, TBE was not considered for any of the patients among the differential diagnoses at admission.

### 3.7. Geographic Distribution of TBEV Reactive Patients

**Figure 4:**
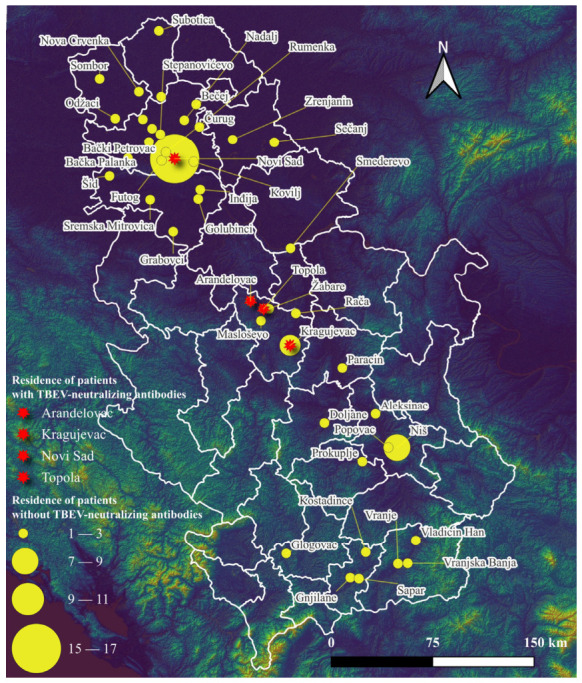
Geographic distribution of patients with TBEV-neutralizing antibodies in Serbia. Proportional symbol map showing the spatial distribution of the municipalities of residence of the 82 patients of this study across Serbia. Symbol size corresponds to the associated count value. The background terrain visualization was obtained with the Copernicus Global Digital Elevation Model (GLO-30), accessed through OpenTopography and processed in QGIS using hillshade and pseudocolor elevation rendering. Red indicates origin of the four patients with TBEV-neutralizing antibodies.

The geographical distribution of patients with TBEV-neutralizing antibodies spanned multiple regions of Serbia, with cases identified in Novi Sad, Aranđelovac, Topola, and Kragujevac (Figure 1 and Figure 4). The data are suggestive of clustering of cases in Šumadija District, which requires further verification (Figure 4).

### 3.8. Association Between Clinical Diagnosis and TBEV Reactivity

TBEV-neutralizing antibodies were observed in patients initially classified as having suspected WNV meningoencephalitis (4/25; 16%), while no seropositive cases were identified among patients with other clinical diagnoses (Figure 1). All of the TBEV-reactive cases were observed among patients diagnosed with viral meningoencephalitis attributed to or suspected to be associated with WNV, consistent with the study hypothesis. No statistically significant association was observed between clinical diagnosis and TBEV exposure (χ^2^ = 9.101, *p* = 0.428). These analyses should be interpreted in a descriptive context given the limited sample size.

## 4. Discussion

In this multicenter retrospective study, TBEV-neutralizing antibodies were detected in a subset of Serbian patients hospitalized with WNV neuroinvasive disease, while TBE was not considered in the differential diagnosis of any patient at admission. These findings indicate exposure to TBEV, although they do not by themselves establish causality in the acute neurological episode. Specifically, four patients showed clear TBEV-neutralizing activity. All the reactive cases occurred among patients initially treated as WNV meningoencephalitis, but where the serologic analysis failed to provide evidence for WNV antibodies. Although TBEV-reactive cases were observed predominantly in older patients and were distributed across several regions of Serbia, these patterns should be interpreted cautiously given the small number of reactive patients and the possibility that individuals may have traveled to areas other than their primary residence.

From an ecological perspective, the detection of TBEV-neutralizing antibodies in the absence of clearly identified foci can be interpreted in the context of the focal nature of TBEV transmission. The apparent discrepancy between the limited identification of confirmed TBEV foci in Serbia and the detection of TBEV-neutralizing antibodies in humans may be explained by the focal and heterogeneous nature of TBEV transmission. TBEV is known to circulate in highly localized microfoci [32], often characterized by specific ecological conditions that facilitate virus maintenance within tick populations and reservoir hosts [21]. These microfoci can remain undetected despite targeted field investigations [21,33], particularly when sampling intensity is limited or not spatially aligned with areas of human exposure [22]. In Europe, TBEV transmission is primarily associated with *Ixodes ricinus*, whose distribution and activity are influenced by environmental and climatic factors, as well as host availability [34]. The maintenance of TBEV in nature depends on complex interactions between ticks and small vertebrate hosts, including rodents, with co-feeding transmission playing a key role [35]. Such ecological dynamics may result in silent virus circulation that is not readily captured through conventional surveillance approaches but may still lead to sporadic human exposure.

In this context, the detection of TBEV-neutralizing antibodies in geographically dispersed patients suggests that TBEV circulation in Serbia may be more widespread than currently recognized, albeit likely occurring in spatially restricted and epidemiologically subtle transmission foci. This highlights the importance of integrating ecological and epidemiological approaches, including targeted vector and reservoir surveillance, to better characterize the distribution and public health relevance of TBEV in the region.

These findings build on previous indirect evidence of TBEV circulation in Serbia [19,20] and provide additional hospital-based evidence that TBEV exposure among patients with compatible neurological syndromes may have been so far overlooked in routine clinical practice. Importantly, this challenge is not unique to Serbia. Underdiagnosis and misdiagnosis of TBE have been reported or suspected in several European regions where TBEV endemicity remains uncertain or insufficiently defined [36,37], particularly because most infections caused by the TBEV European subtype are asymptomatic [38]. At the same time, TBE outbreaks may occur even outside areas traditionally regarded as endemic [39], underscoring the need for sustained surveillance and clinical vigilance beyond well-recognized TBE foci.

Compared with reports from other European regions, the proportion of patients in our cohort with TBEV-neutralizing antibodies was higher than that reported by the International Scientific Working Group on TBE, which identified two TBE cases based on serum and/or cerebrospinal fluid testing via neutralization assay among 233 individuals from France, Belgium, the Netherlands, and Sweden [40]. In this context, Serbia may more closely resemble northwestern Polish regions such as Kujawsko-Pomorskie, Pomorskie, and Zachodniopomorskie, where reported prevalences were 2.0%, 3.0%, and 7.5%, respectively [41]. Although such comparisons should be interpreted cautiously because of differences in study design, patient selection, and laboratory methods, they nevertheless support the plausibility of clinically relevant but underrecognized TBEV circulation in Serbia.

An important observation in our study was that 8.5% (4/47) of patients initially diagnosed as cases of WNV infection showed serological evidence of TBEV exposure. It should also be emphasized that WNV IgG positivity alone does not establish acute WNV infection. In orthoflavivirus-endemic settings, prior exposure and serologic cross-reactivity can complicate interpretation of ELISA-based results, reinforcing the need for confirmatory neutralization testing before assigning a specific acute orthoflaviviral etiology. This finding underscores the importance of incorporating neutralization assays before a specific orthoflavivirus diagnosis is established [28,40]. At present, ELISA remains the main tool used in routine practice for indirect assessment of orthoflavivirus exposure in Serbia; however, in area where multiple orthoflaviviruses co-circulate, its diagnostic performance is limited by serologic cross-reactivity, with reduced specificity and variable sensitivity depending on the timing of sampling [28,38,40]. In such a setting, some patients classified as having WNV infection may have had previously unrecognized TBEV exposure, although the available data do not allow definitive reclassification as acute TBE cases. The detection of a high TBEV neutralization titer (1:640) in one patient further supports the need for confirmatory diagnostic evaluation in similar cases.

This interpretation is clinically plausible because WNV disease and TBE share overlapping neuroinvasive manifestations, and cases of meningoencephalitis occurring during summer and early autumn in Serbia are understandably more likely to be attributed to WNV. Under these circumstances, TBE may remain underconsidered in the differential diagnosis, particularly when a biphasic course is absent, unrecognized, or poorly documented [1]. The principal value of our study is therefore hypothesis-generating and in raising awareness among public health and clinical professionals. It suggests that a TBEV etiology should be more consistently considered in patients with neuroinvasive disease in Serbia, including during the WNV transmission season, and supports the broader use of confirmatory TBEV testing in appropriately selected patients. This interpretation is clinically plausible because WNV and TBE share overlapping neuroinvasive manifestations despite fundamentally different ecological and transmission cycles, with WNV maintained in mosquito–bird systems [42] and TBEV in tick–vertebrate host cycles [35], which may contribute to diagnostic bias when clinical presentation predominates over ecological context [29].

These findings have direct implications for clinical practice, surveillance, and prevention of neuroinvasive arboviral infections in Serbia. TBE should be included in the differential diagnosis of spring, summer, and early autumn central nervous system infections, particularly in patients with suspected WNV meningoencephalitis. In patients with positive or equivocal WNV or TBEV ELISA results, confirmatory testing with neutralization assay or TBEV NS1 ELISA [43,44] should be integrated more systematically to improve etiologic attribution in suspected orthoflaviviral CNS disease [28,40,41]. Such an approach would support more accurate case detection, strengthen national TBE surveillance, and provide a stronger evidence base for public health decision-making, especially regarding vaccine availability, as TBE vaccines are currently not available in Serbia [24,45].

The main strengths of this study include its multicenter design, its clinically targeted retrospective case-finding strategy, and the use of microneutralization in a WNV-endemic setting in which ELISA-based interpretation can be problematic [28]. Several limitations should nonetheless be considered. First, the retrospective design and relatively small sample size limited statistical power and precluded robust subgroup analyses. Second, testing was performed on single serum samples rather than paired acute and convalescent sera, and cerebrospinal fluid was not available for confirmatory analysis. Third, although TBEV-neutralizing antibodies strongly support previous exposure, they do not by themselves prove that TBEV was the cause of the acute neurological episode in every reactive patient. Travel history was not systematically recorded, which limits interpretation of the probable site of TBEV acquisition. Therefore, the municipality of residence should not be considered definitive evidence of local exposure, as infection may have occurred during travel to other regions of Serbia or to established TBEV-endemic areas in neighboring or other European countries. Future prospective studies should include standardized collection of travel history, tick exposure, occupational and recreational risk factors, and vaccination status to more accurately assess the geographic origin of infection. In addition, TBEV-specific IgM testing was not performed. Although microneutralization provides high specificity for confirming TBEV exposure in an orthoflavivirus-endemic setting, detection of neutralizing antibodies in a single serum sample does not distinguish past exposure from acute or recent infection. Therefore, the absence of paired sera, CSF samples, and TBEV-specific IgM results precludes definitive etiologic attribution of the neurological episode to TBEV in individual patients. Accordingly, our findings are best interpreted as evidence of probable underrecognition of TBE in Serbia rather than definitive reclassification of all reactive cases.

Despite the absence of specific antiviral therapy, improved confirmation of TBE cases has important public health implications. Accurate case identification would enable better estimation of disease burden, delineation of geographic risk areas, and differentiation from other neuroinvasive infections, thereby informing targeted vaccination strategies, clinician awareness, and preventive measures such as tick-bite avoidance.

## 5. Conclusions

The detection of TBEV-neutralizing antibodies in 4 of 79 patients hospitalized with suspected WNV neuroinvasive disease or viral encephalitis of unknown etiology provides suggestive evidence of previous TBEV exposure in this patient population. Although these findings raise the possibility that TBE may be underrecognized in Serbia, the small number of positive cases and the absence of paired sera, CSF analysis, TBEV-specific IgM testing, and molecular confirmation preclude definitive etiologic attribution of the acute neurological illness to TBEV. These hypothesis-generating findings support the need for prospective studies incorporating systematic clinical, epidemiological, and laboratory investigation of TBEV infection in Serbia. TBE should therefore be more consistently included in the differential diagnosis of central nervous system infections, and positive or equivocal orthoflavivirus ELISA findings should be followed by confirmatory neutralization testing whenever feasible. Strengthening clinical awareness, diagnostic pathways, and national surveillance will be essential for defining the true burden of TBE in Serbia and for informing evidence-based prevention strategies, including future decisions regarding vaccine availability and administration. These findings should be considered hypothesis-generating and warrant confirmation in prospective, multicenter studies. Such studies should enroll consecutive patients with compatible neuroinvasive syndromes, collect standardized clinical and exposure data, and include paired serum and cerebrospinal fluid samples, with confirmatory neutralization testing alongside diagnostics for other neurotropic pathogens.

## Figures and Tables

**Table 1:** Samples with TBEV-neutralizing antibodies and corresponding TBEV titers.

Sample Code	TBEV Titer
4/21	1:640
5/21	1:20
4/18–19	1:10
14/21	1:10

## Data Availability

All data generated in this manuscript are available in the main text.

## Abbreviations

The following abbreviations are used in this manuscript:

TBE	Tick-Borne Encephalitis
TBEV	Tick-Borne Encephalitis Virus
WNV	West Nile Virus
CNS	Central Nervous System
PHI	Public Health Institute
